# Paradoxical psoriasiform alopecia secondary to secukinumab: a case report^☆^

**DOI:** 10.1016/j.abd.2025.501155

**Published:** 2025-07-01

**Authors:** Inés Segovia Rodríguez, María Castillo Gutiérrez, Fernando Pinedo-Moraleda, Beatriz Aranegui Arteaga

**Affiliations:** a Department of Dermatology. Hospital Universitario Infanta Cristina, Parla, Madrid, Spain; b Department of Pathology, Hospital Universitario Fundación Alcorcón, Alcorcón, Madrid, Spain

Dear Editor,

Psoriasis is a chronic immune-mediated disease that can often be associated with other immune-regulated pathologies. One of these is hidradenitis suppurativa, which has been observed to increase the risk of psoriasis by 3.24 times compared to the general population (95% IC 2.27‒4.62).^1^

The development of biological therapies has enabled the treatment of both conditions. Currently, adalimumab, a monoclonal antibody against Tumor Necrosis Factor-alfa (TNF-α), and secukinumab, an antibody targeting Interleukin (IL) 17A, is approved for the treatment of both diseases. Both are generally safe and well-tolerated, but they may cause adverse effects, such as paradoxical reactions.

We present the case of a 42-year-old woman diagnosed with Hurley II stage hidradenitis suppurativa and long-standing plaque psoriasis affecting the scalp, armpits, inframammary folds, and genital area. She had received multiple systemic treatments for hidradenitis control with insufficient results, including antibiotics (doxycycline and rifampicin-clindamycin combination) and retinoids (isotretinoin and acitretin). Psoriasis lesions were primarily managed with topical treatments, achieving partial control.

To address both conditions, biological therapy with adalimumab was initiated, achieving a good initial response. However, after a year, the treatment lost effectiveness in controlling hidradenitis suppurativa, prompting a switch to secukinumab in December 2022.

Three months after starting secukinumab, the patient experienced a significant worsening of psoriasis, with widespread lesions and two large erythematous and keratotic plaques on the scalp (occipital and parietal regions) with follicular pustules and areas suggesting fluctuation. Differential diagnosis included folliculitis decalvans, dissecting cellulitis, and other causes of cicatricial alopecia (Fig. 1). Biopsies were performed on both plaques, and samples were taken for bacterial and fungal cultures.

**Figure 1:**
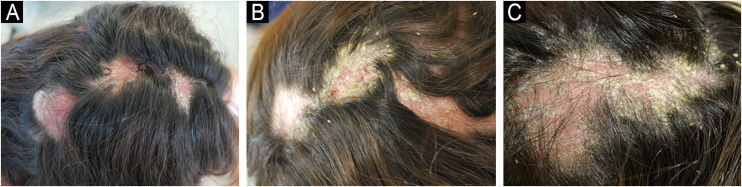
Large plaques on the scalp, in the occipital and parietal regions, initially more erythematous (A), which developed significant hyperkeratosis (B and C).

Given this clinical deterioration, secukinumab was discontinued, and therapy was switched to guselkumab, an IL-23 inhibitor. Additionally, treatment with fluocinolone acetonide and framycetin cream, as well as oral doxycycline for three months, was prescribed.

Histopathological analysis of the biopsies, processed in vertical and horizontal sections, revealed a significant increase in the telogen and vellus indices. Loss and atrophy of sebaceous glands, infundibular dilation with thinning of the follicular epithelium, and a dense perifollicular lymphocytic inflammatory infiltrate were observed without interface involvement. The epidermis exhibited psoriasiform acanthosis and parakeratosis. These histological findings were consistent with psoriatic alopecia (Fig. 2). Cultures were negative.

**Figure 2:**
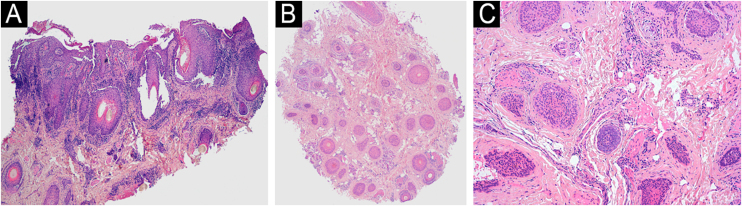
Hematoxylin & eosin histopathology: (A) Vertical section showing psoriasiform hyperplasia, parakeratosis, and a dense lymphoplasmacytic inflammatory infiltrate (×40); (B) Horizontal section showing preservation of follicular units and an increase in the telogen index (×40); (C) Atrophy of sebaceous glands (×100).

After the treatment, the patient showed significant improvement in both conditions, including complete regrowth of alopecic plaques on the scalp, with no signs of psoriasis except for minimal residual scaling (Fig. 3).

**Figure 3:**
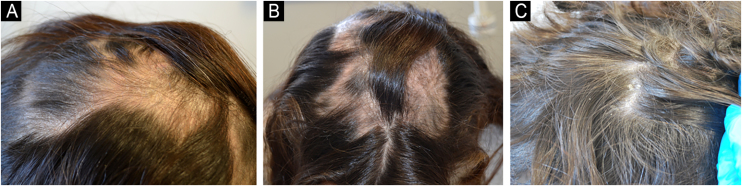
Partial regrowth of psoriatic alopecia plaques after 2 months of guselkumab treatment (A and B). Complete regrowth after 5 months of treatment (C).

Paradoxical reactions involve the onset or worsening of immune-mediated diseases due to the use of monoclonal antibodies. The main paradoxical psoriasiform reactions to biological treatment include palmoplantar psoriasis (42.9%), plaque psoriasis (14.7%) and scalp psoriasis (7%).^2^

Paradoxical psoriasiform alopecia is an emerging and poorly understood type of paradoxical reaction described in recent years. It has been reported more frequently with anti-TNF-α drugs, with few cases documented with IL-17 and IL-12/23 inhibitors.^3^, ^4^, ^5^, ^6^, ^7^ Psoriasiform alopecia can be histologically distinguished from other types of alopecia by sebaceous gland atrophy.^8^ This is a controversial issue, as some authors accept it might be possible to distinguish this condition from primary psoriatic alopecia by the presence of eosinophils and plasma cells in the inflammatory infiltrate. Meanwhile, other authors stand up for the same spectrum of diseases, with similar histopathological findings on skin biopsies.^9^ Cases of permanent alopecia have been described in patients with paradoxical scalp psoriasis.^5^, ^8^

It is recommended to consider discontinuing the causative drug to prevent progression to cicatricial alopecia, especially in severe cases unresponsive to conservative measures.^10^ However, the decision to suspend treatment should be individualized based on the risk-benefit ratio and the severity of the alopecia.

In this case, the temporal sequence, clinical findings, and histopathological features led us to a paradoxical psoriasiform alopecia diagnosis induced by IL-17 inhibitors (secukinumab). This is one of the few cases reported in the literature, demonstrating a very good response after discontinuing secukinumab and initiating guselkumab therapy.

## Financial support

None declared.

## Authors' contributions

Inés Segovia Rodríguez: Original draft preparation, review, editing, read and agreed to the published version of the manuscript.

María Castillo Gutiérrez: Original draft preparation, review and editing, read and agreed to the published version of the manuscript.

Fernando Pinedo Moraleda: Original draft preparation, supervision, review and editing, read and agreed to the published version of the manuscript.

Beatriz Aranegui Arteaga: Conceptualization, methodology, supervision, review, editing, read and agreed to the published version of the manuscript.

## Conflicts of interest

None declared.

## References

[1] Gau S.Y., Preclaro I.A.C., Wei J.C., Lee C.Y., Kuan Y.H., Hsiao Y.P. (2022). Risk of psoriasis in people with hidradenitis suppurativa: a systematic review and meta-analysis. Front Immunol.

[2] Megna M., De Lucia M., Gallo L., Lauro W., Picone V., Fabbrocini G. (2023). Psoriatic alopecia and paradoxical psoriasis induced by adalimumab successfully treated with certolizumab: clinical, trichoscopic, and in vivo reflectance confocal microscopy features. Skin Appendage Disord.

[3] Munera-Campos M., Ballesca F., Carrascosa J.M. (2018). Paradoxical reactions to biologic therapy in psoriasis: a review of the literature. Actas Dermosifiliogr (Engl Ed).

[4] Yajima M., Akeda T., Kondo M., Habe K., Yamanaka K. (2019). Alopecia diffusa while using interleukin-17 inhibitors against psoriasis vulgaris. Case Rep Dermatol.

[5] Tan T.L., Taglia L., Yazdan P. (2021). Drug-induced psoriasiform alopecia associated with interleukin- 17 inhibitor therapy. J Cutan Pathol.

[6] Tirelli L.L., Alfaro A., Citera G., Echeverría C.M. (2022). Nonscarring alopecia secondary to secukinumab. Actas Dermosifiliogr.

[7] Mihailescu M., Cibull T., Joyce J. (2021). Development of drug-induced psoriasiform alopecia in a pediatric patient on ustekinumab. J Cutan Pathol.

[8] Afanasiev O.K., Zhang C.Z., Ruhoy S.M. (2017). TNF-inhibitor associated psoriatic alopecia: diagnostic utility of sebaceous lobule atrophy. J Cutan Pathol.

[9] Doyle L.A., Sperling L.C., Baksh S., Lackey J., Thomas B., Vleugels R.A. (2011). Psoriatic alopecia/alopecia areata–like reactions secondary to anti–tumor necrosis factor-α therapy: a novel cause of noncicatricial alopecia. Am J Dermatopathol.

[10] Osório F., Magro F., Lisboa C., Lopes S., Macedo G., Bettencourt H. (2012). Anti-TNF-alpha induced psoriasiform eruptions with severe scalp involvement and alopecia: report of five cases and review of the literature. Dermatology.

